# How can collective action between government sectors to prevent child marriage be operationalized? Evidence from a post-hoc evaluation of an intervention in Jamui, Bihar and Sawai Madhopur, Rajasthan in India

**DOI:** 10.1186/s12978-018-0552-1

**Published:** 2018-06-28

**Authors:** Venkatraman Chandra-Mouli, Marina Plesons, Alka Barua, Priyanka Sreenath, Sunil Mehra

**Affiliations:** 1 World Health Organization, Geneva, Switzerland; 2 MAMTA Health Institute for Mother and Child, New Delhi, India; 3 Independent expert, Ahmedabad, India

**Keywords:** Child marriage, Multi-sectoral, Adolescent health, India

## Abstract

**Background:**

Although the need for multi-faceted and multi-sectoral approaches to address the multidimensional issue of child marriage is well-acknowledged, there is a dearth of documented experience on the process of implementing and managing such programmes.

**Methods:**

WHO evaluated a district-level, government-led multi-sectoral intervention to address child marriage in Jamui, Bihar and Sawai Madhopur, Rajasthan, implemented by MAMTA Health Institute for Mother and Child (MAMTA). We evaluated the intervention’s design, implementation, monitoring, and outputs and identified key challenges and successes.

**Results:**

Through actions at the state and district levels, the intervention succeeded in creating a cascade effect to stimulate more concerted action at block and village levels, with tangible intersectoral convergence occurring at the village level. The success factors we identified included an experienced partner NGO that was committed to supporting this effort, context-specific design and implementation, and a flexible and responsive approach. However, despite contributing to informal coordination between various stakeholders, the intervention did not succeed in developing a sustained joint-working mechanism at the district level. Shared ownership for prioritization of child marriage across national- and state-level sectors was not established, due in part to lack of directives transcending ministerial/departmental boundaries. Nevertheless, due to its efforts at the district-level, the intervention was able to enlist leadership from the District Magistrates and Child Marriage Prohibition Officers, in line with their duties outlined in the 2006 Prohibition of Child Marriage Act. The challenges we identified included lack of clear directives and institutional support for collaboration, obstacles to monitoring, administrative challenges, differing perspectives on strategy among district leaders, community resistance, and intervention over-commitment.

**Conclusions:**

The findings of this evaluation reveal the potential of multi-sectoral approaches to prevent and respond to child marriage and provide insight into obstacles that affect multi-sectoral coordination. We point to actions that MAMTA could take to strengthen collaboration on this and other initiatives. We also recommend further documentation and evaluation of projects and programmes in this area.

## Plain English summary

To improve knowledge on implementing and managing multi-sectoral approaches to end child marriage, WHO evaluated a district-level multi-sectoral intervention to address child marriage in Jamui, Bihar and Sawai Madhopur, Rajasthan, India, implemented by MAMTA- Health Institute for Mother and Child (MAMTA). We evaluated the intervention’s design, implementation, monitoring, and outputs and identified key challenges and successes. Through actions at the state and district levels, the intervention succeeded in creating a cascade effect to stimulate more concerted action at block and village levels, with tangible intersectoral convergence occurring at the village level. The success factors we identified included an experienced partner non-government organization that was committed to supporting this effort, a context-specific design and implementation, and a flexible and responsive approach. However, despite contributing to informal coordination between various stakeholders, the intervention did not succeed in developing a sustained joint-working mechanism. Shared ownership for prioritization of child marriage across national- and state-level sectors was not established. Nevertheless, the intervention was able to enlist leadership from the key government officers, in line with their duties outlined in the 2006 Prohibition of Child Marriage Act. The challenges we identified included lack of clear directives and institutional support for collaboration, obstacles to monitoring, administrative challenges, differing perspectives on strategy, community resistance, and intervention over-commitment. We point to actions that MAMTA could take to strengthen collaboration on this and other initiatives. We also recommend further documentation and evaluation of projects and programmes in this area.

## Background

Each year, about 12 million girls are married before the age of 18 years [1]. Child marriage is a widely recognized violation of human rights, as it disempowers girls and puts them at risk for lifelong and intergenerational health, economic, and social consequences [2–5].

A third of child brides worldwide live in India, where child marriage remains a persistent issue [6]. Rates in the states of Bihar and Rajasthan are among the highest in the country, with 68 and 58% of young women, respectively, aged 20–24 married before the age of 18, compared to the national prevalence of 47% in 2011 [6–8]. In India, child marriage thrives predominantly in rural areas, where it is perpetrated by a cycle of poverty, lack of education, gender inequality, and social norms [9]. Despite legislation against child marriage since 1929 (which set the still-current legal age for marriage at 18 years for girls and 21 years for boys), most recently in the context of the Prohibition of Child Marriage Act in 2006 and the proposed National Plan of Action to Prevent Child Marriage in 2012 (Table 1) that sought to promote the use of convergent approaches at national, state, and district levels, the country has seen little progress on this issue, especially in terms of dedicated human and financial resources or multi-sectoral coordination [10].

**Table 1:** National Plan of Action to Prevent Child Marriages in India, 2012

Goal: Girls and boys in India are free from child marriage and can realize their full potential and live a life of dignity. Approach: Strategic convergent and multi-dimensional initiatives: • Enforce Prohibition of Child Marriage Act 2006 and related laws and policies that can discourage child marriage. • Improve access to quality education and other vocational opportunities. • Initiate programs that enable community mobilization and outreach to change social norms and attitudes. • Build skills and capacities of adolescent girls and boys. • Collect data, initiate research to inform programming and interventions. • Develop monitoring and evaluation systems for measuring outcomes. • Improve co-ordination, communication and monitoring among those involved in the implementation.	

Child marriage has numerous social and economic drivers and effects. As a result, limited-scale and non-integrated approaches are only narrowly effective [11]. However, convergence approaches, which use multi-sectoral and multi-level institutions to jointly address an issue, have been largely under-utilized in the prevention of child marriage. Work in areas such as nutrition, meanwhile, point to considerations for designing and implementing interventions, including securing political commitment, paying attention to human resources and organizational capacity constraints, and ensuring consensus on selected interventions and roles and responsibilities [12]. However, there is little published knowledge on the use of multi-sectoral approaches to prevent child marriage, especially related to effective strategies for designing, implementing, and evaluating these processes [13].

From 2011 to 2015, a multi-sectoral intervention to address child marriage, aligned with the 2012 National Action Plan, was designed and implemented by MAMTA-Health Institute for Mother and Child (MAMTA) in the districts of Jamui, Bihar and Sawai Madhopur, Rajasthan (Table 2). MAMTA is an Indian NGO working on maternal, newborn, and child health; nutrition; sexual and reproductive health; and communicable and non-communicable diseases in 7 states in India. The intervention’s objective in both states was to support the development of a district-level convergence mechanism to strengthen collaborative efforts to prevent and respond to child marriage at district, block, and village levels. This evaluation does not examine outcomes or effects of the intervention at the individual level, but rather aims to assess the processes used by the intervention to contribute to the formation of collaborative multi-sectoral efforts; the impact of these processes on coordinated action; and the key success factors and challenges encountered by the intervention.

**Table 2:** Intervention Activities

District Level • Support district magistrates/chief executive officers and other district officials in different departments to create cross-departmental coordination committees and convergence plans. • Support the development of a set of monitoring indicators. • Build the capacity of district officials to engage and support block level officials. • Sensitize and engage civil society groups – in particular NGOs and print journalists. Block Level • Sensitize and build the capacity of officials from different departments, and support them in implementing child-marriage prevention activities. Gram Panchayat Level • Reach elected panchayati raj institution leaders, given that they are not always reached because of human and financial resource constraints at the block level.	

## Methods

This evaluation assessed the intervention’s design, implementation, monitoring, and outcomes, with a focus on changes to the administrative and managerial process of preventing and responding to child marriage, to answer the following questions:

1. By what processes did the intervention contribute to the formation of collaborative multi-sectoral efforts to prevent and respond to child marriage? (Table 3, Objectives 1 and 2)
2. Did these processes allow for more coordinated action at the district level? (Table 3, Objectives 3 and 4)
3. Did these processes allow for more coordinated action at block and village levels? (Table 3, Objectives 3 and 4)
4. What were the intervention’s key success factors and challenges? (Table 3, Objectives 3 and 4)

**Table 3:** Data Collection - Objectives, Methods, and Samples [19]

Objectives	Methods	Subjects and samples	
		Jamui	Sawai Madhopur
1. To assess Intervention design	Review of relevant government policy, strategy and documents		
	Review of intervention plans and reports		
	Group discussion with MAMTA field staff	1	1
2. To assess Intervention implementation	Review of intervention plans and reports		
	Group discussion with MAMTA field staff	1	1
	In-depth interviews with government officials	1 State 8 District	3 State 5 District
	Group discussions with block officials	3 (1/block)	3 (1/block)
	Group discussion with nongovernment organization representatives	1	1
	Group discussion with media representatives	1	1
3. To assess Intervention monitoring	Review of intervention plans and reports		
	Group discussion with MAMTA field staff	1	1
	In-depth interviews with government officials	1 State 8 District 3 Block	3 State 5 District 3 Block
	Group discussions with block officials	3	3
4. To assess Intervention outputs	Review of letters of support from the state governments		
	Review of district action plans		
	Review of intervention and district reports		
	Group discussion with MAMTA field staff	1	1
	In-depth interviews with government officials	1 State 8 District 3 Block	3 State 5 District 3 Block
	Group discussions with block officials	3	3
	Group discussion with NGO representatives	1	1
	Group discussion with media representatives	1	1
	Survey of gram panchayat representatives	30	30
	Survey of frontline workers	3 ANM 27 ASHA 30 AWW 30 Teacher	3 ANM 27 ASHA 27 Saathin 30 AWW 30 Teacher

Key: *ANM* Auxiliary Nurse Midwife, *ASHA* Accredited Social Health Activist, *Saathin* Rajasthan Women’s Development Project Worker, *AWW* Anganwadi Worker

### Data collection

Three blocks each in Jamui and Sawai Madhopur were randomly selected for evaluation, out of a total of 10 and 5 blocks, respectively. It was ensured that at least one of the three blocks in each district included demonstration villages, where community activities had been supported by the intervention. Within each block, 30 villages with gram panchayats (unit of local self-government in villages) (approximately 10%) were randomly selected for evaluation. A waiver of ethical clearance was secured from relevant authorities, and formal institutional review board approval was deemed unnecessary due to the nature of the evaluation.

An array of both quantitative and qualitative data collection methods (review of documents, in-depth interviews, group discussions and surveys of frontline workers) were used to gather the necessary information (Table 3). In line with the data collection framework, a tool kit that included semi-structured interview and group discussion guides and structured survey questionnaires was developed, translated, field tested, revised, and finalized between May and July 2015. Data collectors and supervisors were trained in July 2015 and data was collected at the state, district, block, and village levels from July to September 2015.

### Data analysis

With regards to the intervention’s design, implementation, monitoring, and outputs, data was collated and triangulated according to a set of evaluation questions (Table 4). For analysis of the intervention’s design, this data included the rationale and assumptions fundamental to the intervention’s design, the process employed, and the mid-course modifications that occurred. For the intervention’s implementation and monitoring, this data included information on activities assumed at state, district, block, and village levels by MAMTA and other stakeholders. For the intervention’s outputs, this data included outputs (i.e. reach, individual and institutional effects, and actions taken) at state, district, block, and village levels. The intervention’s output information was then analyzed for possible associations with the intervention’s implementation. These four components were then assessed with respect to the four research questions.

**Table 4:** Data Analysis - Evaluation Questions [19]

Evaluation objective	Evaluation questions
1. To assess Intervention design	Content: ▪ What were the components of the intervention? ▪ Did the intervention’s components have the potential to achieve its objectives? ▪ What were the assumptions underlying the intervention’s design? Process: ▪ What was the process used to design the intervention? ▪ Was the intervention’s design revised after implementation began? If so, how and why?
2. To assess Intervention implementation	State level: ▪ What activities were done to secure state-level permission/support? District level: ▪ What activities were done to build awareness and support among key officials in the relevant government departments? ▪ What activities were done to set up a district-level inter-departmental coordination committee/group? ▪ What activities were done to support the development of an inter-departmental district action plan? ▪ What activities were done to build awareness and support among nongovernment organizations and media persons operating in the district? Block level: ▪ What activities were done to build awareness and support among key officials in the relevant government departments? ▪ How was this done through existing systems Village level: ▪ What activities were done to build awareness and support among gram panchayat elected officials?
3. To assess Intervention monitoring	▪ Did the intervention contain a plan to monitor implementation? ▪ Was the monitoring system adequate to track activity implementation and to use information to adjust implementation if needed? ▪ Was implementation monitored? If so, how was this done? ▪ How were the data used? ▪ Did the data contribute to changes in the way in which implementation was done?
4. To assess Intervention outputs	State level: ▪ Whose support was obtained and what level of support was obtained? District/Block level: ▪ Which key gatekeeper officials were reached? ▪ What is the effect of being reached by MAMTA on these officials? ▪ Was a district/block inter-departmental committee set up? If so, is it functional? ▪ Was a district/block action plan developed? If so, what are the contents of the plan and what factors affected the development of the plan? ▪ Is the plan being implemented and what factors affected /affect its implementation? ▪ Is there more concerted action by individual departments? Is there better communication and better synergy between departments? ▪ What was the effect of MAMTA’s outreach with representatives of nongovernment organizations and media persons? Village level: ▪ Were the two key elected officials (Pradhan and Mukhiya) reached? ▪ What is the effect of this on their awareness, motivation and agency – at the individual and collective levels? ▪ Were the ‘marker’ front-line functionaries in the three sectors (Aangan Wadi Workers - AWW, Auxiliary Nurse Midwives - ANM, Accredited Social Health Activists - ASHA, and teachers) reached? ▪ What is the effect of this on their awareness, motivation and agency – at the individual and collective levels?

## Results

This evaluation assessed the findings in response to the four questions posed in the Methods section.

### By what processes did the intervention contribute to the formation of collaborative multi-sectoral efforts to prevent and respond to child marriage?

**Fig. 1:**
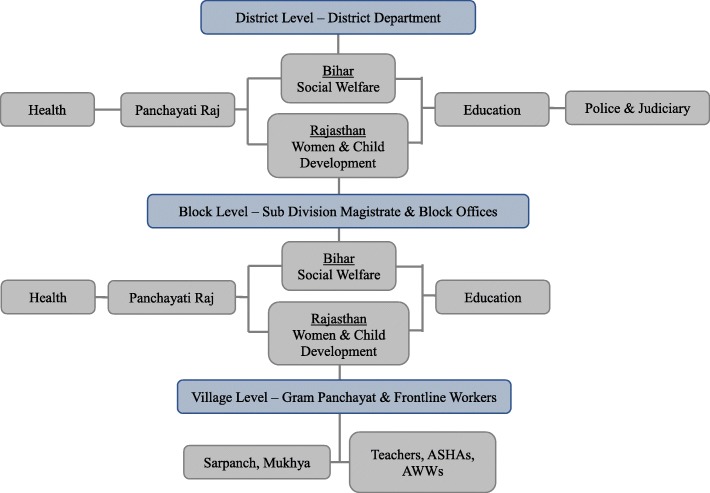
Multi-level actors involved in MAMTA’s efforts

MAMTA took specific actions to engage with different levels of administration to create a cascading downstream effect (Fig. 1). MAMTA secured support for the initiative from relevant authorities at the national and state levels, including the Ministry of Women and Child Development in Rajasthan, and the Department of Social Welfare and its nodal agency dealing with child marriage in Bihar, the Women Development Corporation. Discussions between MAMTA staff and key state government officials then informed selection of implementation districts. At the district level, MAMTA staff conducted a landscape analysis of ongoing activities, and advocated with senior administrative functionaries like the Child Marriage Prohibition Officer (CMPO) and District Magistrate to provide leadership on the intervention. With the senior administrative functionaries’ approval, MAMTA staff then facilitated consultations for consensus-building across departments and pursued joint action planning and the development of corresponding monitoring indicators. They also held advocacy and training sessions for staff of various departments to build capacity and increase awareness of the causes and consequences of child marriage and the relevant laws, policies, and department-specific mandates and responsibilities. To addition to these activities within government sectors, MAMTA staff also sensitized and engaged relevant NGOs to make complementary contributions.

At the block level, MAMTA staff facilitated advocacy efforts directed at key officials to raise the profile of child marriage prevention. Additionally, they conducted and/or facilitated government-led education initiatives and capacity building activities for block level staff and village level frontline functionaries, including Anganwadi Workers (AWWs), Auxiliary Nurse Midwives (ANM), Accredited Social Health Activists (ASHAs), and teachers, ultimately orienting more than two-thirds of frontline workers.

Lastly, at the village level, MAMTA worked to sensitize and orient (or facilitate government-led orientations of) panchayati raj institutions, ultimately orienting about half of gram panchayat representatives.

### Did these processes allow for more coordinated action at the district level?

The intervention contributed to the prioritization of child marriage prevention at the district level. It leveraged the provisions in the 2006 Prohibition of Child Marriage Act that spell out the respective duties of the CMPO and the District Magistrate, along with their specific district-level mandates for preventing child marriage. By raising the profile of child marriage in leadership, the intervention prompted meetings between departmental heads on child marriage prevention and on possible action plans. Key district level officials noted improved understanding of their individual roles and responsibilities. As a result of their commitment to address child marriage, these prominent stakeholders created impetus for lower level coordination.

With the support of MAMTA staff, district level officers formed multi-sectoral coordination and convergence mechanisms to help in the planning, implementation, and assessment of efforts to prevent child marriage. In consultation with district officials, different logistical planning frameworks were established in Jamui and Sawai Madhopur. In Jamui, a district-level Coordination Committee on Prevention of Child Marriage was formed and later integrated with the Child Protection Unit. In Sawai Madhopur, no child marriage committee was established because officials believed existing mechanisms were adequate for discussing the issue. However, all relevant departments did collaborate to address child marriage issues on a single platform.

While integrated departmental plans for preventing child marriage were not established in either district, individual departments in both districts incorporated additional efforts on child marriage prevention into their plans as a result of the intervention. The differing views of district leaders influenced their distinct action plans. For example, the District Magistrate in Jamui focused on education, not prohibition, based on concerns that prohibition could create conflict. Meanwhile, the District Magistrate in Sawai Madhopur valued strict enforcement of legal provisions of the Child Marriage Act, alongside other strategies.

In Jamui, officials participated in MAMTA-initiated meetings designed to discuss convergence approaches to prevent child marriage. Even though there was agreement on the necessity of convergence to address the issue, no efforts were made in conjunction with other departments. In Sawai Madhopur, mixed accounts were given on whether or not coordinated approaches were initiated for child marriage prevention. In both districts, individual departments assumed capacity building and monitoring activities for their respective frontline workers.

### Did these processes allow for more coordinated action at block and village levels?

Although the intervention did not result in extensive district-level coordination to address child marriage, it did succeed in creating impetus and mechanisms for collaboration at the block and village levels.

The 2006 Prohibition of Child Marriage Act also obliges NGOs and panchayati raj members to support the CMPO and District Magistrate in carrying out their duties, which helped to drive the agenda at these levels. MAMTA’s capacity building efforts at the district level also enhanced district officials’ abilities to support and strengthen the work and capacities at the block level. These efforts amplified action at the block level in various independent departments and resulted in a cascade of capacity building activities, down from blocks to villages and gram panchayats to the frontline workers, to improve the system’s ability to coordinate prevention of child marriage.

The support from the block level allowed for improved awareness and action for child marriage prevention among panchayati raj officials. Throughout the implementation of the intervention, MAMTA staff reached over 70% of panchayati raj officials in both districts. Their outreach focused on the sarpanch, mukhiya (elected heads of the panchayati raj), and ward members (elected members of village governance committees) because of their involvement with recording and verifying bride and groom ages for marriage registration and/or disbursement of cash benefits under certain social schemes.

At the village level, there was extensive intersectoral convergence, especially evident by the collaboration among the multi-disciplinary frontline workers. After orientation sessions by MAMTA staff, more than 90% of frontline workers in both districts reported conducting child marriage-related activities in their areas. About 56% of frontline workers in both districts reported conducting these activities at least once a month. They used a variety of entry points (i.e. household visits, Mahila Mandal (women’s group) meetings, Village Health and Nutrition Days, and celebrations during festivals) to conduct a variety of activities (i.e. counseling parents and community members, empowering girls to negotiate with their parents, supporting the establishment of adolescents’ groups, and providing information on the negative consequences of child marriage and its legal implications). About a fifth of frontline workers mentioned sharing child marriage prevention messages during monthly panchayat meetings and Jajam Baithaks (action planning sessions). Additionally, more than 60% reported counselling adolescents and their family members of child marriage prevention.

### What were the intervention’s key success factors and challenges?

Building on the findings to questions 2 and 3, we identified key success factors and challenges of the intervention.

### Key success factors

#### An experienced partner NGO that was committed to supporting this effort

MAMTA staff were critical in designing a suitable programme, monitoring and adapting the strategy as the intervention progressed, and persevering through challenges to maximize impact at the village level. MAMTA built on its previous experience with convergence processes in maternal health and nutrition, and understood the importance of assuming a technical assistance perspective, rather than an implementer perspective. As a result, MAMTA acted as a facilitator for change, supported capacity building and implementation where needed, and contributed to the formation of mechanisms that could continue operating once the intervention funding ended. For example, MAMTA staff engaged a high-level experts group, consisting of national policy-makers, donors, and NGOs who had also contributed to the proposed National Plan of Action to End Child Marriage, to design the intervention. It also provided technical support and advocated with district officials in both districts, who then had the capacity to mirror these activities at the district and village levels. Furthermore, MAMTA played a facilitative role in sensitizing and orienting frontline staff, and supporting them to carry out outreach at the community level.

The approach used by MAMTA was also critical for the intervention’s success. They were keen advocates who utilized emotional appeal, organizational mandate arguments, a positive development perspective, and an emphasis on positive impact to persuade officials to improve and expand efforts on child marriage prevention. These strategies played an important role in convincing officials of the validity and value of child marriage measures.

#### A context-specific design and implementation

Recognizing the institutional and programmatic differences and distinct social and health nuances of the two districts, the intervention was sensitive to the unique and dynamic context of each district in the intervention design and implementation. For example, while Rajasthan had designated staff for child marriage prevention through state mandates, Bihar had no designated officials and the intervention faced competing demands. Additionally, in Jamui, a district level Coordination Committee on Prevention of Child Marriage was established, but was later integrated into the newly formed Child Protection Unit. Meanwhile, Sawai Madhopur officials decided to rely on existing mechanisms and developed a joint district level action plan. Lastly, different authorities in the two states facilitated the cross-departmental monitoring system for district level activities: the Department of Women and Child Development in Rajasthan and the Department of Social Welfare in Bihar. MAMTA worked with the states independently to design and manage a programme that fit both the needs and capabilities of each district.

#### A flexible and responsive approach

The design of the intervention was both flexible and responsive, so as to complement the actions at state, district, block, and village levels to strengthen efforts to prevent child marriage. This flexibility allowed MAMTA to respond to unanticipated changes to the intervention during its implementation. For example, just as MAMTA built off their previous experience with convergence approaches in maternal health and nutrition, they emphasized a learning agenda in Bihar and Rajasthan. After reviewing the first year’s monitoring data, the district level was found to be more involved with planning and strategy compared to the block level, which was more focused on implementation. Thus, in the second year, the intervention’s Advisory Committee decided to invest more heavily at the block and village levels. As a result, programmes were initiated to promote prevention of child marriage with community stakeholders in 10 villages. By monitoring intervention activities and learning from the evidence of their evaluations, MAMTA was able to shape and reshape their strategy to achieve best results.

Additionally, MAMTA staff was accommodating to requests from district officials, in line with their facilitative role. In Sawai Madhopur, a senior district official requested that MAMTA’s staff train women in panchayati raj institutions on child marriage prevention to strengthen outreach activities. In Jamui, the District Magistrate requested that MAMTA conduct workshops for all block level officials in the district. These requests reflected capacity and resource constraints, along with recognition among officials about the need for partnerships with other agencies/NGOs and/or resource mobilization within departments to carry forward the directives. Additionally, these requests demonstrate the willingness to act if provided with adequate support in terms of resources and capacity building. In deference to their desire for mutual partnerships, MAMTA accommodated a number of additional undertakings outside their initial scope of work.

### Key challenges

#### Lack of clear directives and institutional support for collaboration

Despite the 2006 Prohibition of Child Marriage Act’s call for greater awareness of and attention to child marriage, India has no policy that outlines the roles, responsibilities and accountability of different sectors to prevent and respond to child marriage. The result of this within each sector was evident in our evaluation. There was a general lack of clarity on governmental directives, and many officials were either unaware of their responsibilities or were skeptical of the utility of child marriage prevention activities. The result of this on collaboration was also evident. Collaboration was dependent on the initiative of an individual, which hinders effective and sustained coordination with true buy-in from multiple sectors.

Flowing from this, institutional support for a dedicated District Child Marriage Committee was lacking, as evidenced by its absence in Rajasthan and its subsequent merging with the Child Protection Unit in Bihar. Despite acknowledging that convergent efforts would be helpful, no activities were planned or carried out jointly. Thus, the intervention did not succeed in helping to create a unified cross-departmental plan for addressing child marriage.

#### Obstacles to monitoring

No state mandate for monitoring child marriage exists, which made it difficult to create integration within government management information systems and to persuade officials to monitor relevant activities. If a state mandate existed, there would have been opportunity to streamline monitoring of child marriage. An additional major issue involved challenges at the national and state levels for integrating additional relevant indicators in the existing departmental monitoring systems at the district and block levels. Because this was not feasible, a parallel monitoring system was developed; departmental staff were not motivated to collect and report additional information; and its use was not sustained beyond 3 months.

#### Administrative challenges

There were several administrative challenges that posed obstacles to individual departments’ intended actions related to child marriage. Frequent staff turnover and transfers required additional sensitization, training, and capacity-building efforts, and this need was exacerbated by a lack of institutional memory and clear mandates/directives. Additionally, intervention staff and personnel at all levels faced competing priorities, especially in Bihar where there were no state-mandated designated staff for child marriage prevention activities. Lastly, resource allocation was problematic, as exemplified by the insufficient government budget to print Information, Education, and Communication (IEC) materials, and investments did not transcend departmental boundaries.

#### Differing viewpoints of district leaders on strategy and community resistance

Some officials expressed doubt towards MAMTA’s strategy and provided examples of comparative approaches for other issues, such as polio. They highlighted the need for comprehensive and repetitive education to reinforce understanding among communities and officials about issues associated with child marriage. This disagreement was exacerbated by the later lack of follow-up at the district level by MAMTA, especially in the context of high staff turnover among CMPOs and District Magistrates, leading others to question the utility of the intervention. Similarly, there were cases of diametrically opposed opinions about the appropriate strategy for addressing child marriage, for example, emphasizing education or prohibition.

Although media personnel and NGO representatives asserted that the government would need to work in cooperation with them and other civil society groups to end child marriage, elected officials and frontline workers reported that media representatives and NGOs did not have a strong presence and did not work cohesively in their districts.

Additionally, there were difficulties implementing MAMTA activities at the village levels due to resistance from communities and limited support from government officials. As many as 40% of Panchayati Raj members surveyed in Jamui and 24% in Sawai Madhopur reported challenges discussing child marriage in their communities. Many cited hesitancy about reporting child marriage cases, fearing anger and retribution from communities.

#### Intervention over-commitment

A major challenge was lack of supplemental staff upon expansion of efforts at the block and village levels. The intervention was obliged to divert available human and financial resources from the district to lower levels of the system, and MAMTA staff attempted to continue providing technical support and guidance at the district, block, and village levels. However, the intervention staffing numbers did not match the increase in demand. MAMTA staff was overextended, and there was a noticeable lack of follow up at the district level.

## Discussion

In both Bihar and Rajasthan, the intervention succeeded in raising the profile of child marriage and increasing the efforts of individual departments working on the issue. However, the extent of convergent action differed by government level. At the district level, despite contributing to some informal coordination between different departments, the intervention did not aid in developing a sustained joint-working mechanism. Similarly, external factors such as competing priorities, lack of clear governmental directives, and fear of negative community reactions prevented some district and block level officials from initiating action. NGOs and civil society were already engaged in activities to prevent child marriage, but collective/coordinated action between them and the district level government departments did not ensue. At each progressively lower level, however, concrete inter-sectoral convergence occurred. Because block level officials were engaged and sensitized, they were then able and willing to collaborate with other departments to augment activities by Panchayati Raj institutions and frontline workers, with support from MAMTA. To an even greater extent, frontline workers from health, education and community development and the media undertook new initiatives to prevent child marriage. Factors that contributed to success included an experienced partner NGO that was committed to supporting this effort, a context-specific design and implementation, and a flexible and responsive approach. Meanwhile, key challenges included lack of clear directives and institutional support for collaboration, obstacles to monitoring, administrative challenges, differing viewpoints on strategy, community resistance, and intervention over-commitment.

Because of the multiplicity of drivers of child marriage, multi-sectoral and convergent approaches are understood to be key to create sustained declines in child marriage [14, 15]. With regards to evaluations of multi-sectoral convergent approaches, previous initiatives revealed similar challenges to those of this intervention. An initiative in Afghanistan designed to mobilize efforts and strengthen local government structures to respond to gender issues such as child marriage had some success in addressing child marriage, but did not establish collaborative links between community based organizations, government sectors, and NGOs [16]. Similarly, a project implemented in Yemen that aimed to reduce the prevalence of child marriage through a multifaceted approach resulted in modest effects in reducing child marriage. However, its attempts to strengthen capacities met limited success and the coordination efforts were constrained to the national level [17]. A programme in Bihar, India used a multi-sectoral convergence approach focused on building capacity, instituting policy and institutional changes, and improving coordination networks [18]. It was able to successfully build partnerships with all levels of government and was able to enhance the capacity, communication, and a level of engagement at the state, district, and block levels [18].

These findings have a number of implications for initiatives aimed at increasing intersectoral collaboration to address child marriage. First, government policies and strategies should call for multi-sectoral collaboration, allocate resources, issue clear directives for multi-sectoral collaboration mechanisms – with specific roles and responsibilities for the key departments to be involved – and define measures to assess progress. Further national policies and strategies should describe the complementary contributions that different government departments would need to make based on an analysis of the drivers of child marriage, evidence on effective interventions to address these drivers, and an analysis of the government apparatus at the different levels. At the same time strategies should provide room for tailoring this generic guidance to local needs. Second, efforts should be made to advocate with and to support state, district and block level officials to step up their efforts to prevent child marriage. Coordination mechanisms should include a well-designed and resourced component to foster collaboration with NGOs and other civil society bodies. Finally, interventions should apply a long-term vision, employ periodic reviews and joint action planning/problem-solving processes with officials at district and block levels, and use benchmarks to assess progress.

This evaluation had several limitations. First, this is an evaluation of efforts to bring about district-level convergence in two districts within two states in India. Interventions working to contribute to this objective in other settings will thus need to consider the unique political and social context. Second, MAMTA was involved in a diverse set of actions to contribute to the formation of a collaborative system to prevent child marriage; the multiplicity of actions makes it difficult, however, to analyze whether certain individual actions were more effective than others. Third, the intervention brought about whatever changes it did over a three-year period: Given additional time, its effects on individuals and institutions may well have been greater. One of the strengths of the evaluation was the diversity of data collection methods used and the ability to triangulate their findings to corroborate and support overall findings. Another was stakeholder participation in data collection, which allowed for a comprehensive understanding of points of commonality or differences within the single system.

## Conclusions

The findings of this evaluation reveal the potential of multi-sectoral approaches to prevent and respond to child marriage and provide insight into obstacles that affect multi-sectoral coordination. By highlighting the challenges and successes of the intervention, this evaluation provides a better understanding of what it takes to translate the vision of multi-sectoral coordination to prevent child marriage into reality. MAMTA’s findings, which align with previous evaluations, reinforce the need to continue to evaluate and research processes to improve coordination and convergence in addressing child marriage. Programmes already in place require institutional directives to support their efforts and adequate allocation of financial, time, and staff resources to address common systematic administrative challenges. Additionally, as governments continue to enhance their sectoral actions and their intersectoral coordination to address child marriage, broader efforts need to be made to transform social norms to end harmful traditional practices like child marriage.

## Abbreviations

ANM: Auxiliary Nurse Midwives
ASHA: Accredited Social Health Activists
AWW: Anganwadi Worker
CMPO: Child Marriage Prohibition Officer
IEC: Information, Education, and Communication
MAMTA: MAMTA- Health Institute for Mother and Child

## References

[1] UNICEF (2018). Press release: 25 million child marriages prevented in the last decade due to accelerated progress, according to new UNICEF estimates.

[2] UNFPA (2015). Girlhood, not motherhood: Preventing adolescent pregnancy.

[3] Ganchimeg T, Ota E, Morisaki N (2014). Pregnancy and childbirth outcomes among adolescent mothers: a World Health Organization multicountry study. BJOG.

[4] Savadogo A, Wodon Q (2017). Impact of child marriage on intimate partner violence across multiple countries. Education global practice.

[5] Wodon QT, Male C, Nayihouba KA (2017). Economic impacts of child marriage: global synthesis report.

[6] UNICEF (2014). Ending child marriage: progress and prospects.

[7] Srinivasan P, Khan N, Verma R, Giusti D, Theis J, Chakraborty S (2015). District-level study on child marriage in India: What do we know about the prevalence, trends and patterns?.

[8] UNICEF (2015). The state of the World’s children 2015.

[9] UNICEF, UNFPA (2017). Ending child marriage in India.

[10] Abbhi A, Ayakumar K, Raj M, Padmanabhan R (2013). Child marriages in India: an insight into law and policy.

[11] ICRW, Girls not Brides (2016). Taking action to address child marriage: the role of different sectors.

[12] Pelletier DL, Frongillo EA, Gervais S, Hoey L, Menon P, Ngo T (2012). Nutrition agenda setting, policy formulation and implementation: lessons from the mainstreaming nutrition initiative. Health Policy Plan.

[13] Travers E, Moudouthe F (2015). Lessons learned from selected national initiatives to end child marriage.

[14] Hervish A, Feldman-Jacobs C (2011). Policy brief: Who speaks for me? Ending child marriage.

[15] Lee-Rife S, Malhotra A, Warner A, Glinski AM (2012). What works to prevent child marriage: a review of the evidence. Stud Fam Plan.

[16] Gandhi K, Krijnen J (2006). Evaluation of community-based rural livelihoods programme in Badakhshan, Afghanistan.

[17] Pedersen K, Mukred A, Qaid E (2008). Evaluation of “Integrated Action on Poverty and Early Marriage” Programme in Yemen.

[18] Kamath R, Malviya A, Conilleau J, Heiberg T, Nirmal A (2010). Child marriage: robbing children of innocence. Good practices in preventing child marriage, Bihar.

[19] Chandra-Mouli V, Barua A, Igras S (2016). Strengthening collective response of the government to end child marriage through a district level convergence approach in Jamui, Bihar and Sawai Madhopur, Rajasthan, India.

